# Playing surface, match outcomes, and formation usage patterns in Major League Soccer

**DOI:** 10.1371/journal.pone.0358364

**Published:** 2026-09-18

**Authors:** John David Johnson II, Michael Hales, Joshua Pitts

**Affiliations:** 1 Kennesaw State University, Kennesaw, Georgia, United States of America; 2 Mississippi College, Clinton, Mississippi, United States of America; Universidade Federal de Goias, BRAZIL

## Abstract

Playing surface is a routine contextual factor in professional football, yet its association with tactical behavior and match outcomes remains unclear. The aim of this retrospective observational study was to examine whether playing surface is associated with starting formation, performance metrics, and match outcomes in professional football. Using 6,764 team-match observations from 3,383 Major League Soccer (MLS) matches (2017–2024; 6,761 team-match observations with identifiable surface), this study evaluated how surface type (grass, turf, hybrid) is associated with formation usage, performance metrics, and match outcomes. Team-level data were analyzed using chi-square tests, ANOVA, and logistic regression models incorporating surface type, match location, and their interaction. Surface type was not significantly associated with match outcomes (χ²(4) = 7.71, p = 0.103; Cramer’s V = 0.034), suggesting similar competitive outcomes across modern playing surfaces. However, differences in league-wide formation distributions and aspects of attacking performance were observed across surface categories. Formation usage varied significantly by surface (χ², p < 0.001), with the 4–3–3 more common on grass and the 4–2–3–1 more prevalent on turf. Modest differences in goal scoring were observed, while possession remained largely unchanged. Home advantage was strong overall and did not differ significantly between grass and turf; a significant hybrid-by-location interaction was observed, although the hybrid category represented a single venue and should not be generalized. These findings indicate that playing surface was not meaningfully associated with competitive outcomes but was associated with differences in league-wide formation distributions and selected performance metrics. Because home surface is closely tied to club identity and the observational design cannot establish tactical adaptation, formation findings should be interpreted as descriptive associations rather than evidence that teams changed formations in response to surface conditions.

## Introduction

Playing surface is a routine contextual factor in professional football, with teams in Major League Soccer (MLS) regularly competing on natural grass, synthetic turf, and hybrid fields. Coaches and analysts frequently adjust tactical preparation based on surface conditions, yet it remains unclear whether these adjustments are meaningfully associated with match outcomes. This study examines whether playing surface is associated with playing dynamics, and whether these differences translate into competitive results. Understanding this relationship is relevant to performance analysis, opponent scouting, and interpretation of contextual variation in elite competition, while recognizing that observational league data cannot establish whether teams adapt tactically because of surface conditions.

A longstanding assumption in both sport science and applied practice is that these differences are meaningfully related to match outcomes. However, empirical evidence has been inconsistent, with several studies suggesting that modern synthetic surfaces do not substantially alter competitive balance [1,2]. This raises the question: if playing surface is associated with differences in the mechanics and structure of play, why does it not appear to be associated with how matches are ultimately decided?

Addressing this question requires distinguishing between performance processes and performance outcomes. Surface-related changes in ball speed, traction, and player–surface interaction may be associated with how teams organize tactically and execute attacking actions, even if these effects do not propagate to match results. From a performance perspective, playing surface can be understood as an environmental constraint associated with collective behavior without necessarily determining competitive outcomes [3,4].

Turf provides a relatively uniform and mechanically consistent surface that produces faster ball speeds and more predictable bounce characteristics compared with grass, which may exhibit variability in firmness, traction, and ball-roll behavior. Beyond mechanical properties, recent case analyses have also noted environmental and thermal differences between synthetic and natural pitches that may be associated with differences in match tempo and player perception [5]. In response, players and coaches adjust their technical and tactical approaches. Surface-related differences in movement demands and activity profiles have been reported across competitive settings, and recent evidence indicates that artificial turf is associated with increased match running demands compared with natural grass [6,7].

Two comprehensive studies [8,9] examined player–surface interactions using electromyography, wearable metabolic systems, and motion analysis. The studies demonstrated significant differences in muscle activation, recruitment patterns, and neuromechanical load across field types, with higher activation in the gastrocnemius and hamstrings observed on turf, while grass facilitated more consistent quadriceps recruitment. Similar surface-dependent running variations have also been observed in elite women’s football [10], supporting the broader generalizability of surface-related performance modulation. Collectively, these findings suggest that athletes adopt movement strategies that are contingent on surface properties, underscoring the importance of surface-specific preparation.

Prior work suggests that exposure to varied playing environments is associated with changes in how teams organize and respond to novel task constraints [11]. Players may also engage in different risk-taking behaviors in relation to perceived surface safety, with evidence suggesting that grass surfaces may encourage more aggressive or high-risk actions relative to turf [7]. Despite these biomechanical, physiological, and perceptual differences, the extent to which surface-related patterns translate into meaningful differences in match outcomes remains unclear. Accordingly, the present study addresses a critical gap between process-level differences and outcome-level stability in professional football performance.

While prior studies have examined surface-related differences in isolated performance metrics, it remains unclear whether surface-related variation in team behavior reflects meaningful performance advantages or simply alternative expressions of equivalent competitive strategies at scale. To our knowledge, this study provides a league-scale evaluation of how playing surface is associated with team formation, performance metrics, and competitive outcomes in professional football, while explicitly distinguishing descriptive surface-associated patterns from causal or adaptive interpretations.

Based on an ecological dynamics framework and prior literature on player-surface interactions, we formulated the following hypotheses. **Primary hypothesis:** (1) Match outcomes, defined as win, draw, or loss, would not differ across surface types, reflecting maintained competitive balance. **Secondary hypotheses:** (2) starting-formation distributions would vary by surface; (3) attacking output, but not possession, would differ across surfaces; (4) surface × location interactions would emerge, building on the well-established expectation that home advantage is present overall; and (5) goal margins would remain small across surfaces despite any observed tactical or performance variation.

## Materials and methods

### Study design and data source

This study employed a retrospective observational design, analyzing data from MLS matches. Match-level and team-level data (match results, goals for/against, possession percentage, and starting formations) were obtained from FBref.com (Sports Reference LLC) [12]. The dataset included 3,383 MLS matches played across multiple seasons (2017–2024). Most matches are represented by two team-match records, one for each participating club. Because a small number of source records lack a corresponding opponent-side record, the cleaned dataset contains 6,764 team-match observations representing 3,383 distinct matches, identified using the home-team record. Unless otherwise specified, sample sizes reported as team-match observations (n = 6,764; n = 6,761 with identifiable surface) reflect this team-specific record structure, whereas sample sizes reported as matches (n = 3,383) reflect deduplicated distinct games based on the retained home-team record. S1 Table provides a complete accounting of every sample size reported in the analyses and the exclusion criteria underlying each. S1 Fig maps the derivation of every sample size reported in the manuscript, (A) the identifiable-surface branch feeding Tables 1–3; (B) the two match-level branches used for Hypothesis 1 and the overall home-advantage test; (C) the two independent paths that both arrive at n = 3,383; and (D) derivation of the 6,562 formation-transition sample. Matches for which pitch surface could not be identified using surface indicator variables or venue-level fallback coding were excluded from surface-specific analyses. Each match record contained team-specific metrics, performance indicators, and environmental descriptors, including pitch surface classification.

**Table 1:** Match outcomes and performance metrics by surface type and match location (home vs. away).

Surface–Location	n	Win%	Draw%	Loss%	GF (M ± SD)	GA (M ± SD)	GD (M)	Poss% (M ± SD)	PPG
**Grass – Home**	2532	49.72	24.13	26.15	1.78 ± 1.31	1.21 ± 1.10	0.57	52.01 ± 8.60	1.73
**Grass – Away**	2431	25.67	24.27	50.06	1.21 ± 1.11	1.77 ± 1.31	−0.56	48.18 ± 8.54	1.01
**Turf – Home**	673	50.97	25.11	23.92	1.72 ± 1.31	1.11 ± 1.05	0.61	52.55 ± 8.57	1.78
**Turf – Away**	782	25.58	25.06	49.36	1.14 ± 1.05	1.71 ± 1.30	−0.57	48.50 ± 8.72	1.02
**Hybrid – Home**	130	38.46	28.46	33.08	1.56 ± 1.10	1.52 ± 1.29	0.04	53.49 ± 8.20	1.44
**Hybrid – Away**	120	35.83	30.83	33.33	1.58 ± 1.33	1.54 ± 1.11	0.04	47.21 ± 7.92	1.38

***Note.*** Excludes the 2020 neutral-site “MLS is Back” tournament (92 observations removed); final analytic sample n = 6,668. Ns differ from Table 2 because Table 1 is restricted to team-match observations with complete surface, location, outcome, and performance data.

**Table 2:** Performance metrics by surface.

Surface	n	Goals For (M ± SD, 95% CI)	Goals Against (M ± SD, 95% CI)	Possession (M ± SD, 95% CI)
**Grass**	5030	1.50 ± 1.25 (95% CI [1.47, 1.53])	1.49 ± 1.24 (95% CI [1.45, 1.52])	50.14 ± 8.79 (95% CI [49.90, 50.38])
**Turf**	1477	1.41 ± 1.21 (95% CI [1.35, 1.48])	1.44 ± 1.23 (95% CI [1.37, 1.50])	50.37 ± 8.93 (95% CI [49.91, 50.82])
**Hybrid**	254	1.59 ± 1.23 (95% CI [1.44, 1.75])	1.55 ± 1.23 (95% CI [1.40, 1.70])	50.47 ± 8.72 (95% CI [49.40, 51.54])

*Note.* Values are means ± SD. n reflects team-match observations (one row per team per match); the 6,761 team-match observations with identifiable surface correspond to 3,383 distinct matches. Goals For/Against means (n = 4,991 grass; 1,457 turf; 254 hybrid) exclude 59 team-match observations decided by penalty shootout, whose recorded goal tallies were not usable in these calculations; Possession means were based on available observations (grass n = 5,029; turf n = 1,477; hybrid n = 254). Pairwise standardized mean differences (Cohen’s d) ranged from 0.07–0.15, indicating substantial overlap across surfaces.

**Table 3:** Formation usage by surface.

Formation	Grass %	Turf %	Hybrid %
**4–2–3–1**	41.34	45.77	38.98
**4–3–3**	17.52	12.39	16.54
**4–4–2**	8.55	6.77	16.54
**3–4–3**	9.68	7.99	6.30
**3–4–1–2**	3.84	6.03	1.57
**4–1–4–1**	3.14	3.32	11.81
**3–5–2**	3.00	2.84	1.97
**4–1–2–1–2**	2.76	2.78	1.18
**4–3–1–2**	2.41	2.44	0.79
**5–3–2**	2.05	2.84	0.39
**Other (<2%)**	5.71	6.84	3.94

*Note.* “Other” includes the following low-frequency formations (each <2% within every surface category): 3-1-2-2-2; 3-1-4-2; 3-2-1-2-2; 3-2-2-1-2; 3-2-2-2-1; 3-2-3-2; 3-3-2-2; 3-3-3-1; 3-5-1-1; 4-1-3-2; 4-2-2-1-1; 4-2-2-2; 4-3-2-1; 4-4-1-1; 4-5-1; and 5-4-1. Formation percentages were calculated among observations with valid formation data: grass n = 5,029, turf n = 1,477, and hybrid n = 254 (total n = 6,760).

This study utilized publicly available match-level and team-level data and did not involve human subjects as defined by institutional review guidelines. Therefore, ethics approval and informed consent were not required.

### Variables

The main outcome variables included the match result, expressed as win, loss, or draw from the home team’s perspective; the performance metrics, which covered goals for, goals against, and possession percentage (goals for is used throughout as the primary indicator of attacking output/performance); the tactical indicator, reflected in formation usage such as 4–3–3, 4–2–3–1, or 5–3–2; and playing surface classification. Additional covariates included match location (home or away) and team identity. Team-level effects refer to individual club identities rather than tiered, ranking-based, or categorical classifications of team quality.

Team formations were defined as the officially reported starting formations listed in MLS match records. Formation data were obtained directly from league match documentation and reflect the tactical structure declared at kickoff. In-match tactical adjustments, phase-specific shapes (e.g., attacking versus defensive organization), and transient positional shifts were not coded. When formation changes occurred after kickoff, only the starting formation was retained for analysis to ensure consistency across matches. Accordingly, formation data should be interpreted as reflecting initial team formation rather than full-match tactical dynamics. This approach prioritizes methodological consistency across a large multi-season dataset while capturing variation in officially reported starting team shape. Starting formations were classified based on the recorded formation field and grouped into structural families for supplementary analyses (see S2 Table). Hybrid pitch systems vary in synthetic content depending on manufacturer and installation with most commercial systems reported at approximately 90–95% natural grass and 5–10% synthetic fiber by composition. The single hybrid venue represented in this dataset uses SISGrass hybrid technology, documented by the venue operator as approximately 95% natural grass and 5% synthetic fiber [13]. Since this study’s hybrid category is represented by a single venue and system, findings for hybrid surfaces should not be generalized to hybrid systems with substantially different synthetic-to-natural ratios (see Limitations).

Accordingly, the present analysis focused on match-level performance indicators consistently available across all seasons, rather than event-level metrics such as passing length, spatial distribution, or pass success rates. This choice reflects a deliberate emphasis on league-scale inference over event-level granularity, while acknowledging that event-level analyses may provide additional mechanistic insight into surface-related performance differences. Unless otherwise specified, descriptive performance statistics are reported at the team-match level (i.e., one observation per team per match). In contrast, inferential analyses were conducted at the match level. Analyses requiring surface- and location-specific stratification were restricted to observations with complete outcome, possession, location, and surface information.

Because hybrid surface observations were relatively sparse, hybrid matches were excluded from highly stratified formation-family models to avoid unstable estimates but retained in primary outcome analyses, where estimates were explicitly labeled exploratory and accompanied by confidence intervals.

### Surface classification

In this study, the term “turf” refers exclusively to third-generation (3G) synthetic turf systems incorporating rubber infill and designed to replicate key mechanical properties of natural grass. All MLS turf pitches during the study period utilized modern 3G technology. The term “grass” refers to natural grass playing surfaces maintained according to elite professional standards and evaluated under Fédération Internationale de Football Association (FIFA)-recommended quality protocols, acknowledging that FIFA does not provide formal certification for MLS natural playing surfaces but instead promotes continuous validation through standardized testing procedures. The term “hybrid” refers to natural grass surfaces reinforced with synthetic fibers or structural elements. In line with FIFA guidance, hybrid surfaces are classified within the broader category of natural playing surfaces and are evaluated using natural surface testing protocols rather than certified under synthetic turf standards. Playing surface classification was conducted at the stadium level using official league documentation and historical venue records. Each team’s surface designation was linked to its primary home stadium and updated by season when documented stadium changes or resurfacing occurred (see S3 Table). Matches played at neutral, temporary, or indeterminate venues, or for which surface type could not be confidently resolved, were excluded from surface-based analyses. Cross-validation of surface indicators, venue records, and home/away surface codes was performed to minimize misclassification. In the present study, however, hybrid surfaces were analyzed as a separate category to preserve potential differences in surface mechanics and environmental constraints relevant to tactical behavior. Because detailed pitch-level mechanical measurements (e.g., stiffness, traction, or ball-roll metrics) were not uniformly available across venues and seasons, surface classifications were used as practical ecological proxies reflecting typical differences in playing environment.

During 2020, MLS suspended its regular schedule and staged the “MLS is Back” tournament entirely at a single neutral site (ESPN Wide World of Sports Complex, Kissimmee, FL.). This tournament consisted of 95 team-match observations (47 home-designated records, 48 away-designated records; the asymmetry reflects two clubs, FC Dallas and Nashville SC, that withdrew from the tournament due to COVID-19 outbreaks, whose forfeited group-stage matches are recorded only from their opponents’ perspective). These matches were retained in surface-based analyses, for which match location is immaterial, but excluded from all analyses involving match location (home vs. away). All other postseason matches in the dataset, across all seasons, were hosted at the higher seeded team’s home stadium under MLS’s standard single elimination playoff format and therefore reflect genuine home-venue conditions. These data were retained in all analyses alongside regular-season data.

### Statistical analysis

Descriptive statistics were used to summarize the distribution of match outcomes and performance metrics by surface type. Chi-square tests of independence were used to assess associations between surface type and categorical outcomes (win/loss/draw and formation use). Because most matches contribute two team-match records, one for each participating club, the formation chi-square analysis should be interpreted as a descriptive test of association among team-match observations rather than a fully independent match-level test. To assess sensitivity to this dependence structure, formation-surface associations were also examined separately for home and away observations, with the home-only analysis providing a one-observation-per-match comparison.

T-tests and one-way ANOVA were used to compare continuous variables, including possession and goals scored, across the different surface categories. For more comprehensive analysis, logistic regression models were used to evaluate the probability of a win (1 = win, 0 = draw or loss) as a function of surface type, match location, and their interaction. A secondary possession × surface model used binary logistic regression with win status as the dependent variable and possession percentage, surface type, and their interaction as predictors. Season was included as a fixed effect. The overall interaction was evaluated using a likelihood-ratio test, with a team-clustered robust Wald test used as a sensitivity analysis. The baseline model took the form:


$$\begin{array}{c} \text{Logit }(P({\text{Win}}_{\text{ij}}=\text{1})=\beta_{\text{0}}+\beta_{\text{1}}{\text{Surface}}_{\text{ij}}+\beta_{\text{2}}{\text{Location}}_{\text{ij}}+\beta_{\text{3}}{\left(\text{Surface }\times \text{ Location}\right)}_{\text{ij}} \end{array}$$

where ${\text{Win}}_{\text{ij}}$ denotes the binary outcome (win = 1), *Surface* represents playing surface type, *Location* indicates home versus away status, β_0_ is the model intercept, β_1_ is the coefficient for the main effect of surface, β_2_ is the coefficient for the main effect of location, and $\beta_{\text{3}}\;$ is the coefficient for the interaction between surface and location. Interaction terms tested whether surface effects differed by venue. Extended specifications included season as a fixed effect and team-clustered (Huber–White robust) standard errors to account for repeated observations within clubs across years. A sensitivity model excluding hybrid-surface observations was fitted using the same specification to assess whether the grass-versus-turf comparison was robust to removal of the small hybrid subgroup. Multicollinearity among predictors was assessed using variance inflation factors (VIF), with no evidence of problematic collinearity observed. In these logistic regression models, inference focused on population-average associations rather than team-specific random effects. Beyond these primary population-average models, exploratory team-level variation analyses were conducted using mixed-effects logistic regression models (random intercept for team) to account for repeated observations within clubs across seasons. These models estimated whether individual clubs exhibited surface-specific deviations in win probability (e.g., relatively stronger performance on turf than grass) while retaining the same fixed-effect structure for surface, location, and their interaction, with season retained as a fixed effect across all specifications.

Formation trends were analyzed using frequency distributions and chi-square tests to determine if formation usage significantly differed between surface types. Because the number of matches differed across surface types, formation usage was evaluated using both raw frequencies and percentages normalized to the total number of games played on each surface. In addition, statistical procedures that account for unequal group sizes were used across analyses, including chi-square tests based on expected frequencies, unbalanced ANOVA, and logistic regression. Additional analyses included the interaction between surface type and possession on match outcomes, surface-specific performance by formation, and team-level variation to playing surface.

Analyses were conducted using Python (pandas 2.3.3, stats models 0.14.5) or IBM SPSS Statistics v31 and verified with appropriate assumptions checks such as expected cell counts and normality. Normality of continuous variables was assessed using visual inspection of distributions and Shapiro–Wilk tests where appropriate, and homogeneity of variance was evaluated using Levene’s test. A significance level of α = 0.05 was adopted throughout. All statistical tests were two-tailed. Effect sizes and 95% confidence intervals were reported where appropriate, and summary statistics were rounded to two decimal places, with additional precision reported for very small values when necessary for accurate interpretation. Primary hypotheses (match outcome by surface, goals scored by surface, formation usage by surface, and surface × location interaction) were evaluated without adjustment, while exploratory analyses (including detailed formation-family comparisons and interaction tests) were evaluated using the Benjamini–Hochberg false discovery rate (FDR) correction. Given the large sample size, emphasis was placed on the magnitude and practical relevance of observed effects rather than statistical significance alone. Accordingly, effect sizes and confidence intervals were interpreted as primary indicators of meaningful differences, with p-values used to contextualize statistical uncertainty. Given the observational design and use of aggregated match-level data, these models are intended to identify population-level associations rather than establish causal relationships or mechanistic explanations of performance. Outliers were not removed, as all observations represented valid match-level data. Missing data were handled using case-wise exclusion for analyses requiring complete information (e.g., surface–location stratification), as described in Table 1.

Two team-match records were removed during data preparation prior to analysis: one duplicate entry (erroneously entered twice for the same match) and one record for a 2020 match that was cancelled and never played; neither reflects an actual observed team-match event. Because most matches contribute paired team-match records reporting the same outcome from opposite perspectives, the primary test of match outcomes by surface type (Hypothesis 1) was conducted at the match level, using one retained record per distinct match. The surface-location stratified analysis (n = 6,668) additionally excludes the 2020 neutral site tournament matches described above, since match location is not meaningful for those observations. Unlike the goals for/against statistics reported elsewhere, which excluded shootout-decided matches entirely, this surface-location breakdown instead retains these matches using the regulation-goals count preceding the shootout notation (e.g., “0” from “0(1)”), so that the location-stratified breakdown would not lose additional observations; this difference in handling is noted here for transparency.

## Results

### Match outcome distribution by surface type

Across team-match observations with identifiable surface type (n = 6,761), surface distribution was as follows: 5,030 on grass, 1,477 on turf, and 254 on hybrid surfaces (Table 2). Because most matches contribute paired team-match records reporting the same result from opposite perspectives, the association between surface type and match outcome was tested at the match level using one retained record per distinct match (*n* = 3,383) Chi-square analysis of match outcomes by surface type yielded no statistically significant association (χ²(4) = 7.71, *p* = 0.103, with a very small effect size (Cramer’s V = 0.034), indicating no meaningful evidence that match outcomes differed by surface type.

When match location (home vs. away) was incorporated, the outcome distribution differed significantly across surface–location combinations (χ²(10) = 507.22, p < 0.001; Cramer’s V = 0.195), indicating a strong venue effect with modest variation across surfaces. The 2020 MLS is Back tournament contained 95 team-match observations overall. Three observations lacked identifiable surface classification; therefore, 92 neutral-site observations were excluded from the identifiable-surface surface-location analysis.

### Performance metrics

Performance metrics by surface type are summarized in Table 2. Analysis of goals and possessions showed that teams playing on grass averaged 1.50 goals for and 1.49 goals against, those competing on turf averaged 1.41 goals for and 1.44 goals against, and teams on hybrid surfaces averaged 1.59 goals for and 1.55 goals against (Table 2). Comparable analyses were conducted for goals conceded and possession percentage in addition to goals scored. One-way ANOVA confirmed a statistically significant difference in goals scored across surface types (F(2, 6699) = 3.70, *p* = 0.025), with effect size η² = 0.001, indicating minimal variance in scoring attributable to surface type. This analysis, and the goals for/against statistics in Table 2, excluded 59 team-match observations decided by penalty shootout, whose recorded goal tallies included non-numeric shootout notation (e.g., “0(1)”) not usable in the goals-based analyses (grass n = 4,991; turf = 1,457; hybrid = 254). Although statistically significant, this effect explains less than 0.1% of the variance in goals scored, indicating that surface type contributes minimally to offensive output relative to other match determinants. Because goals scored were analyzed at the team-match level, opposing teams’ goal totals are inherently linked within matches. Accordingly, this result should be interpreted cautiously, and match-level sensitivity analyses may provide a more robust assessment of whether scoring environments differ across surface categories. Pairwise standardized mean differences indicated substantial overlap in scoring distributions across surfaces, with effect sizes for grass vs. turf (d = 0.07), grass vs. hybrid (d = 0.08), and turf vs. hybrid (d = 0.15). Goal margin (goals for minus goals against) did not differ meaningfully by surface type (one-way ANOVA: F(2, 6699) = 0.28, p = 0.759, η2 < 0.001).

**Fig 1:**
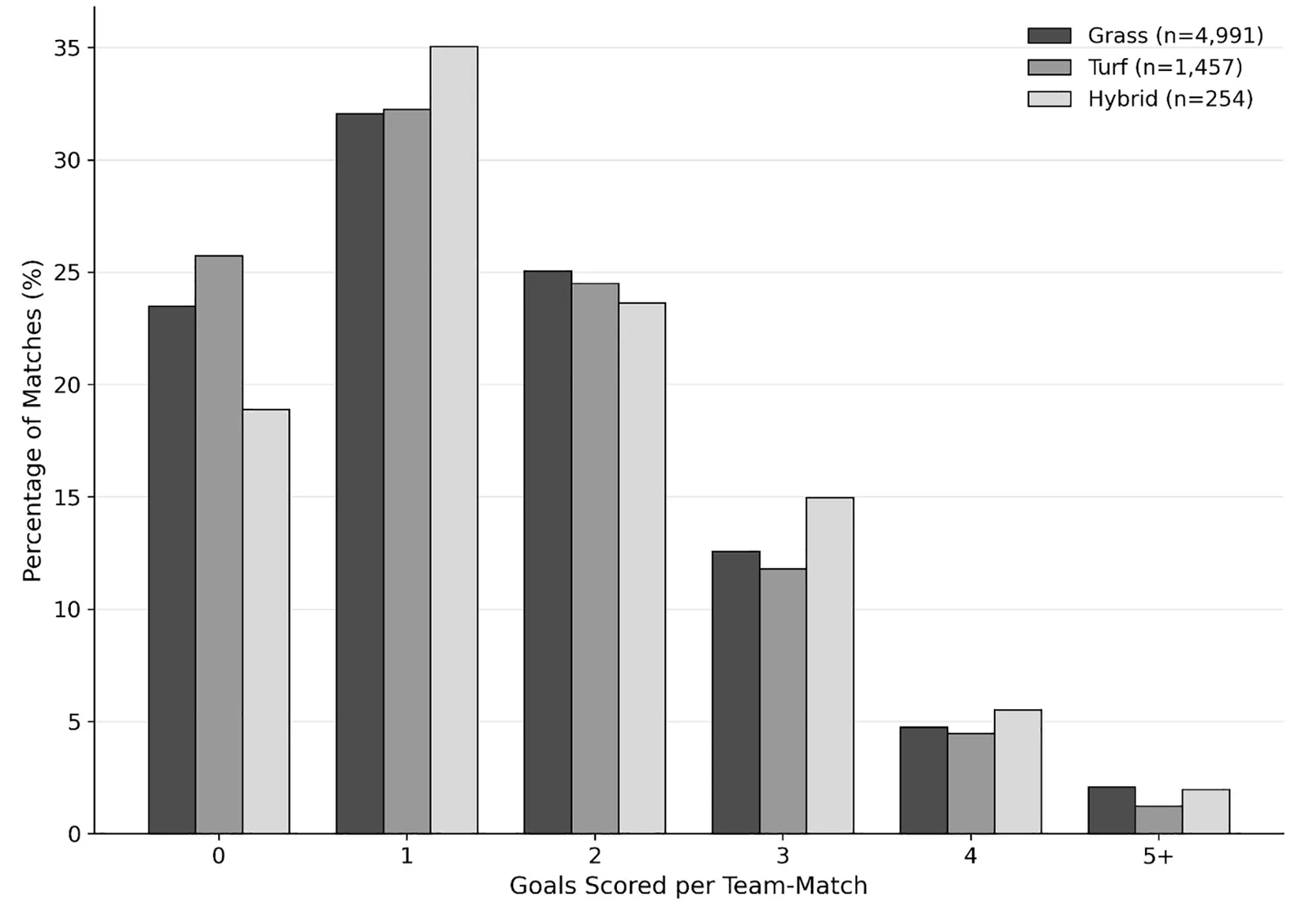
Goals scored per match by playing surface. Boxes represent the median and interquartile range (IQR); whiskers indicate 1.5 × IQR. Circles denote group means, and vertical error bars represent 95% confidence intervals.

Hybrid surfaces accounted for a comparatively small number of team-match observations (n = 254), with further reductions after stratification by match location. Accordingly, hybrid related estimates should be interpreted as exploratory. Mean goals scored on hybrid surfaces were accompanied by wide confidence intervals relative to grass and turf, reflecting greater sampling uncertainty. Visual inspection of the distribution of goals scored by surface type (Fig 1) indicates substantial overlap across surfaces, with hybrid fields showing a modestly higher median but no pronounced separation in overall scoring distributions.

### Formation usage

Formation usage by surface type is presented in Table 3. Formation analysis revealed modest variation in starting formation use across surface types. The most frequently used formation on turf was 4–2–3–1, while grass and hybrid surfaces showed higher relative use of 4–3–3 and 4–4–2 variants. Chi-square testing confirmed a significant association between surface type and formation choice (χ²(50) =191.84, *p* < 0.001, Cramer’s V = 0.12). These findings indicate a modest league-level association between surface category and reported starting formation distributions across surface types rather than wholesale restructuring of team shape.

Supplementary stratified analyses indicated that the formation-surface association was substantially stronger among home teams than among away teams. Because home surface is closely linked to club identity in MLS, these findings suggest that persistent club-level tactical tendencies may contribute to the observed association. Consistent with this interpretation, teams were not significantly more likely to change formation when playing surface changed between consecutive matches. The observed formation patterns should therefore be interpreted primarily as league-level associations rather than direct evidence of surface-driven tactical adaptation.

Percentage-normalized frequencies further clarified these differences in formation usage across surfaces. The 4–3–3 accounted for a higher proportion of matches on grass (17.5%) than on turf (12.4%), indicating a modest surface-associated difference in its use. In contrast, the 4–2–3–1 remained the dominant system across all surfaces and showed particularly high relative use on turf (45.8%), compared with 41.3% on grass and 39.0% on hybrid pitches. Hybrid surfaces also showed observed differences in formation distribution, including elevated use of the 4–4–2 (16.5%) and several three-center-back formations.

A relatively large number of formation variants were observed across seasons. To enhance interpretability, closely related formations were additionally grouped into broader structural families (e.g., back-four systems, back-three systems) for comparison (see S2 Table).To evaluate whether surface-related formation patterns reflected longer term tactical evolution in MLS, seasons were grouped into early (2017–2020) and recent (2021–2024) periods and formation-family analyses were repeated within each window. Surface-family associations were present in both eras, although effect sizes were attenuated in the later period. A log-linear test of the family × surface × era interaction was significant, indicating that the surface–formation relationship changed in magnitude and/or composition across eras while remaining detectable in both periods (2017–2020: Cramer’s V = 0.116; 2021–2024: Cramer’s V = 0.074).

To examine whether surface–formation associations changed within seasons as teams accumulated experience, matches were split into early and late halves within each team–season. Formation family remained associated with surface type in both early and late matches, although effect sizes were smaller later in the season. A log-linear test of the family × surface × phase interaction was not significant, indicating that the overall structure of the association did not differ meaningfully between early and late phases (Early: Cramer’s V = 0.085; Late: Cramer’s V = 0.066). These results indicate that the family-surface association remained stable across early and late phases of the season. A separate, team-level analysis examined whether teams were more likely to change their starting formation between consecutive matches when the playing surface also changed. Among 6,562 consecutive within-season, within-team match transitions, formation changed 36.9% of the time when the surface also changed, compared with 35.4% when the surface stayed the same, a small difference that was not statistically significant (χ^2^(1) = 1.46, p = 0.227).

### Surface-related analyses

Possession was also examined in relation to match outcome. Possession percentage was weakly but significantly negatively associated with winning on grass (*r* = −0.09, *p* < 0.001) and turf (*r* = −0.08, *p* = 0.001), while no association was observed on hybrid surfaces (*r* = −0.00, *p* = 0.95). In a season-adjusted logistic regression model, the overall possession × surface interaction was not statistically significant (likelihood-ratio χ²(2) = 1.87, *p* = 0.392). A sensitivity analysis using team-clustered robust standard errors produced the same conclusion (Wald χ²(2) = 2.38, *p* = 0.305). These results indicate no convincing evidence that the association between possession and winning differed across surface types.

An interaction between surface type and match location was observed. Match outcomes and performance metrics stratified by surface type and match location are presented in Table 1. Considered on its own, match location was strongly associated with match outcome; home teams won 49.6% of matches compared with 26.0% for away teams (χ^2^(2) = 485.80, p < 0.001; Cramer’s V = 0.27), confirming a robust home-pitch advantage across the dataset as a whole, independent of surface type. Logistic regression analysis indicated that home advantage was a strong predictor of winning on grass, the reference surface (OR = 2.87, 95% CI [2.43, 3.37], p < 0.001; team-clustered robust standard errors). The main effect of turf relative to grass was small and not significant (OR = 0.99, 95% CI [0.87, 1.14], p = .922), and the turf × location interaction was also not significant (OR = 1.06, 95% CI [0.81, 1.38], p = .677), indicating that home advantage did not differ meaningfully between turf and grass. The hybrid × location interaction, by contrast, was significant (OR = 0.39, 95% CI [0.27, 0.57], p < 0.001), indicating a smaller home-advantage effect on the hybrid surface; since hybrid is represented by a single venue in this dataset, this pattern more plausibly reflects a team-specific effect rather than a generalizable property of hybrid surfaces (see Limitations). A joint Wald test of both interaction terms was significant (χ^2^(2) = 25.79, p < 0.001), driven entirely by the hybrid term; a sensitivity model restricted to grass and turf only (excluding hybrid) produced the same substantive pattern. Home location remained strongly associated with winning (OR = 2.87, 95% CI [2.44, 3.38], *p* < 0.001), whereas the turf × location interaction was not statistically significant (OR = 1.06, 95% CI [0.82, 1.37], *p* = 0.679), providing no evidence that the magnitude of home advantage differed between grass and turf.

To further examine the interaction between playing surface and match location, home win rates differed only modestly between grass and turf surfaces (Table 1), while away performance was more consistent across surfaces. Goals scored and goals conceded followed a similar pattern, with grass and turf surfaces showing comparably large home–away differentials (goal difference swinging by roughly 1.15 goals between home and away in both cases), in contrast to a much smaller differential on hybrid surfaces (about 0.03).

Possession values varied minimally across surfaces and locations, indicating limited variation in ball-control metrics across contexts. Exploratory team-level analyses were also conducted. When home and away matches are considered separately, this apparent surface-associated advantage is substantially reduced. Supplementary mixed-effects logistic regression models (random intercepts for team) should be interpreted with this confound in mind. Sensitivity analyses in which hybrid surfaces were alternatively collapsed with grass or turf yielded qualitatively similar surface × location and formation-family patterns, indicating that the primary findings were not driven by the small hybrid subsample.

A secondary, exploratory analysis examined whether the magnitude of home advantage changed over the study period. Home advantage declined significantly across seasons: the home–away win-percentage gap fell from 32.8 percentage points in 2017 to 13.1 percentage points in 2024 (Table 4). A linear trend across seasons was significant (r = −0.86, p = 0.006), and a logistic regression testing the interaction between match location and season (treated as a continuous predictor) confirmed that this decline was statistically robust (likelihood-ratio test, p < 0.001).

**Table 4:** Home advantage by season.

Season	n	Home Win %	Away Win %	Gap (pts)
2017	354	54.8	22.6	32.8
2018	391	53.7	25.3	28.4
2019	404	53.5	25.0	28.5
2020	254	49.6	27.2	22.4
2021	455	47.3	25.3	22.0
2022	472	47.7	27.3	20.3
2023	503	48.5	23.5	25.0
2024	503	44.3	31.2	13.1

*Note.* Calculated using deduplicated distinct match records (n=3,383), where Home n represents the total number of distinct matches within each season, and Away Win % is structurally equal to Home Loss %. The 2020 values exclude neutral-site observations from the “MLS is Back” tournament. Gap = Home Win % minus Away Win %. Linear trend across seasons: r=−0.86,p=0.006. Logistic regression location × season interaction: likelihood-ratio p<0.001.

## Discussion

Consistent with Hypothesis 1, match outcomes were not meaningfully associated with surface type. Starting-formation distributions and some attacking-performance indicators varied across surface categories, but these associations were small in magnitude and should be interpreted as descriptive league-level patterns rather than evidence of direct tactical adaptation.

This distinction between process-level variation and outcome-level stability is central to interpreting surface effects in professional football: playing surface may remain a relevant contextual factor when evaluating tactical organization in different match environments, even though it was not associated with a systematic competitive advantage at the league level. Surface type was associated with differences in starting-formation distributions at the league level, with the 4–3–3 disproportionately favored on grass, the 4–2–3–1 dominant across all surfaces but especially prevalent on turf, and hybrid surfaces showing different observed formation distributions including elevated use of the 4–4–2 (Table 3). These differences were statistically significant and held after normalizing the unequal number of matches played on each surface.

Formation-family analyses suggested that the observed association was not limited to a small number of individual formation labels. Because formation families reflect broader structural choices regarding defensive organization, midfield density, and width [14], these findings may indicate variation in overall team structure rather than isolated formation preferences. However, because formation usage was more strongly associated with surface among home teams than away teams, the observed patterns should still be interpreted cautiously and may reflect club-level tactical preferences as well as surface-related influences.

Although these patterns are potentially consistent with surface-related tactical considerations, the present dataset cannot determine why certain formations were more common on particular surface categories. Because formation usage was more strongly associated with surface among home teams than away teams, and because home surface is closely tied to club identity, the observed pattern may reflect stable club-level tactical preferences in addition to any direct influence of surface conditions. Future studies incorporating event-level or team-adjusted analyses could help distinguish between these explanations.

Beyond individual formations, these findings speak to the broader concept of style of play as it relates to surface conditions. Starting formation is only a coarse indicator of style of play, and the present data cannot determine whether the observed formation distributions reflect adaptation to surface conditions, club-specific tactical philosophies, or a combination of both. However, these observed differences in formation distribution did not correspond to a difference in how style was translated into results: the relationship between possession and winning was weakly negative on grass and turf, with no statistically significant evidence that the association differed by surface. Together, these patterns suggest that surface may be associated with observable differences in team shape without altering the underlying relationship between style and competitive outcomes. Since style of play encompasses dimensions beyond starting formation, including buildup patterns, pressing intensity, and tempo that were not directly measured in the study, these findings should be regarded as a preliminary structural perspective on style rather than a comprehensive characterization.

With respect to attacking formation, surface type was associated with differences in aspects of attacking performance. Goals scored varied significantly across surfaces, with hybrid fields seeing slightly higher average goals than grass or turf. However, goals conceded and possession remained statistically unchanged. This mixed pattern partially supports Hypothesis 3, as not all performance metrics were affected, although offensive output showed measurable differences. These findings are consistent with context-dependent patterns of positional organization during match play [3,4]. Recent modeling work further emphasizes that context-specific performance variables are necessary for explaining goal-directed behavior under varying task constraints [11]. Findings involving hybrid surfaces should be interpreted cautiously given the limited number of observations relative to grass and turf. Although hybrid fields exhibited slightly higher mean goals scored and different formation frequencies, confidence intervals were wide and the study was not powered to draw firm conclusions regarding hybrid-specific effects.

Another important finding involved the interaction between surface type and home pitch status. Home advantage was strong overall (OR = 2.87, 95% CI [2.43, 3.37, p < 0.001] on grass; team-clustered robust standard errors), confirming that playing at home substantially increases the probability of winning on grass and turf, consistent with contemporary multi-league analyses demonstrating persistent venue effects in elite football [15]. The exception was the hybrid surface, where the home-advantage effect was significantly attenuated relative to grass; given that this surface is represented by a single venue in the dataset, we interpret this as a team-specific pattern rather than evidence that hybrid surfaces generally reduce home advantage. Surface × location differences were detectable in the full outcome distribution (win/draw/loss; analysis excluded the 2020 neutral-site tournament matches), indicating that the relationship between venue and results varies modestly by surface. However, in population-average win-only logistic regression models the surface × location interaction was not statistically robust. Taken together, these results indicate that home advantage in MLS is clearly present and context-dependent, but that surface type does not consistently or robustly amplify or diminish its magnitude. Recent econometric analyses further suggest that the magnitude of home advantage scales with crowd size and officiating dynamics, reinforcing the contextual nature of venue-related effects [16]. A recent meta-analysis also reported no meaningful video assisted referee (VAR)-associated reduction in goals-based home advantage, suggesting that venue effects remain robust even under enhanced officiating review [17]. Thus, Hypothesis 4 receives partial support, with interaction effects appearing context-dependent rather than uniformly strong. These findings do not replicate earlier reports suggesting that turf systematically amplifies home advantage [18], as surface-related differences in home performance were modest and not robust across model specifications. Our results are more closely aligned with MLS specific research showing minimal home advantage on turf fields [2].

Beyond its association with surface, home advantage in this dataset declined substantially and significantly across the 2017–2024 study period, with the home–away win-percentage gap falling by more than half (Table 4). This decline was independent of surface type. While the present data cannot identify a specific cause, this pattern is broadly consistent with reports of eroding home advantage in other leagues over comparable periods and suggests that season or era may be at least as important a contextual factor as playing surface when interpreting venue effects in MLS.

Finally, although average goal differences between surface types were minimal and statistically nonsignificant, MLS matches are typically decided by narrow margins, often just one goal, meaning that small surface-related effects may be relevant in specific competitive contexts where performance margins are small. Goal margin showed little evidence of variation across surfaces in this dataset (one-way ANOVA: F(2, 6699) = 0.28, p = 0.759, η² < 0.001), consistent with Hypothesis 5. From an applied perspective, these findings suggest that context-specific tactical adjustments may support performance consistency without necessitating wholesale strategic change.

Across analyses, statistically significant findings were generally associated with small effect sizes, indicating that surface-related differences reflect modest shifts rather than large structural competitive advantages. These estimates support interpreting surface type as a contextual performance constraint rather than a primary driver of competitive imbalance, consistent with prior work demonstrating surface-dependent differences in ball mechanics, player movement, and tactical behavior [1,7─^9^].

Notably, this study took place during a period where 3G turf dominated MLS stadiums, making our results highly relevant to current surface technologies. Earlier findings based largely on second generation (2G), or other lower-quality synthetic fields may no longer be applicable [1]. Within the context of this MLS dataset, the findings suggest that modern turf surfaces were associated with similar match outcomes to grass while still being associated with subtle differences in gameplay and tactics.

In practical terms, the present findings suggest that playing surface remains a relevant contextual variable when evaluating team performance and tactical organization. From a performance perspective, variation in constraints such as playing surface can be associated with differences in positional organization [19]. Because surface type was not associated with large or uniform shifts in competitive outcomes, wholesale tactical redesign based solely on surface is unlikely to be warranted; however, context-specific adjustments may be beneficial. Possession was weakly negatively associated with winning on both grass and turf, with no evidence of meaningful surface moderation. This pattern suggests that possession share alone should not be interpreted as a direct indicator of tactical effectiveness, particularly because match state and other contextual factors were not modeled. This pattern, while counterintuitive, is consistent with prior findings that losing teams often exhibit higher ball possession than winning teams, possibly reflecting a tendency for teams trailing in a match to accumulate possession while attempting to force an equalizer [20].

Overall, the present findings integrate competitive balance, tactical variation, and contextual performance patterns into a coherent interpretation of surface effects in MLS: playing surface functions as a contextual constraint shaping structural decision-making and marginal performance, without determining competitive outcomes.

### Limitations

This study has several notable limitations. First, the absence of individual player tracking and event-level data restricts our ability to examine micro-level movement behaviors and more granular tactical actions, such as acceleration profiles, sprint frequency, passing patterns, and spatial distribution. Although match-level metrics such as goals scored, goals conceded, and possession percentage are widely used indicators of performance, surface-related effects may also be reflected in finer tactical behaviors not captured in the present dataset. Future research incorporating player tracking or event-level data may provide more detailed insight into how surface conditions could be related to movement patterns, spacing, and transition dynamics during match play, particularly with respect to execution-level metrics such as passing efficiency and expected-value models.

Second, while the dataset includes 3,383 matches (6,764 team-match observations) across multiple seasons, it is limited to team-level observations, preventing analysis of position-specific or individual responses to surface conditions. For example, the extent to which different positional roles adjust movement patterns or tactical responsibilities across surfaces remains unclear.

Additionally, the hybrid-surface category in this study is represented by a single venue and hybrid system (approximately 95% natural grass, 5% synthetic fiber). Hybrid systems industry-wide vary in synthetic context, and findings reported in the study for hybrid surfaces should not be assumed to generalize hybrid systems with significantly different compositions. Relatedly, this study did not have access to venue-level field-testing data (e.g., impact attenuation, traction, or ball-roll measurements) for the sampled stadiums; such data are not made publicly available by venue operators, so the extent to which grass, turf, and hybrid surfaces were held to directly comparable performance standards across venues could not be independently verified. Third, although statistical models controlled for surface type, formation, and home advantage, the observational design limits causal inference. Because surface type is not randomly assigned across MLS venues, observed associations may reflect structural scheduling, venue characteristics, and contextual factors rather than direct surface effects. In real-world league competition, playing surface cannot be randomly assigned in a true experimental sense because it is embedded within fixed venue and scheduling structures; accordingly, stronger causal inference would likely require quasi-experimental designs, simulated match settings, or natural experiments involving venue surface changes. Consequently, surface-related differences should be interpreted as context-dependent performance patterns rather than definitive causal mechanisms.

An additional limitation concerns interpretation of the observed formation-surface association. Because MLS clubs generally play their home matches on fixed surface types, surface category is closely associated with club identity. Supplementary analyses indicated that the formation-surface association was substantially stronger among home teams than away teams, and teams were not significantly more likely to change formation when moving between surface types. Consequently, the observed formation distributions should be interpreted as league-level associations and may reflect persistent club-level tactical tendencies as well as, or instead of, direct adaptation to playing surface.

Finally, psychological and perceptual factors, including player confidence, surface preference, and familiarity, were not directly measured but may be associated with performance outcomes. These variables warrant further investigation using survey-based or experimental approaches. Future research employing controlled or quasi-experimental designs may help clarify how surface-related variations are associated with performance processes without necessarily determining competitive outcomes.

## Conclusion

This study indicates that playing surface was not meaningfully associated with MLS match outcomes from 2017–2024. Match outcomes were similar across grass, turf, and hybrid pitches, and effect sizes for outcome-level analyses were small. Some process-level indicators, including goals scored and starting-formation distributions, varied across surface categories, but these differences were modest and should be interpreted cautiously.

The formation findings are best understood as descriptive league-level associations rather than direct evidence of surface-driven tactical adaptation. Because home surface is closely tied to club identity and the formation-surface association was concentrated among home observations, observed formation patterns may reflect persistent club-level tendencies, venue context, or other unmeasured factors as well as, or instead of, direct responses to playing surface.

Overall, the findings support the conclusion that modern MLS playing surfaces are not associated with meaningful differences in competitive outcomes. Playing surface may remain a useful contextual variable for performance analysis, but it should be interpreted alongside club identity, venue conditions, opponent characteristics, season, and scheduling factors rather than treated as an independent driver of tactical behavior or competitive balance.

## Supporting information

S1 Fig Derivation of sample sizes reported throughout the manuscript. (TIF)

S1 Table Complete accounting of every sample size (n) reported in the manuscript. Rows are ordered to follow the logical exclusion chain from the full dataset down to each analysis-specific sample. Two values recur with different meanings: 3,383 denotes both the total distinct-match count and, separately, the identifiable-surface and Hypothesis-1 match-level counts, all of which are numerically equal in the corrected dataset; 254 denotes both the total hybrid-surface team-match count and the hybrid subset with usable (non-shootout) goals data, which are equal because no hybrid-surface match in the dataset was decided by penalty shootout. Formation-family Cramer’s V values reported for the era-split and season-phase analyses are not included here, as they derive from the formation-family grouping rather than from the sample-size chain summarized above.
(DOCX)

S2 Table Classification of individual formations into structural formation families. Recorded starting formations were grouped into broader structural families for descriptive purposes. All inferential statistical analyses were conducted using the original formation classifications.
(DOCX)

S3 Table Home playing surface for each Major League Soccer club in the dataset. Surface classification reflects the primary home stadium used by each club during the study period. Teams retained the same surface designation across seasons unless a documented stadium or surface change occurred.
(DOCX)

## References

[1] Barnett V, Hilditch S. The effect of an artificial pitch surface on home team performance in football (soccer). J R Stat Soc Ser A: Stat Soc. 1993;156(1):39. doi: 10.2307/2982859

[2] Trombley MJ. Does artificial grass affect the competitive balance in major league soccer? JSA. 2016;2(2):73–87. doi: 10.3233/jsa-160020

[3] Araújo D, Davids K, Hristovski R. The ecological dynamics of decision making in sport. Psychol Sport Exerc. 2006;7(6):653–76. doi: 10.1016/j.psychsport.2006.07.002

[4] Davids K, Araújo D, Vilar R, Renshaw I, Pinder R. An ecological dynamics approach to skill acquisition: implications for development of talent in sport. Tal Dev Excell. 2013;5(1):21–34.

[5] Cumberbatch IS, Richardson L, Grant-Bier E, Kayali M, Mbithi M, Riviere RF, et al. Artificial turf versus natural grass: a case study of environmental & playability differences. Sustainability. 2025;17(14):6292. doi: 10.3390/su17146292

[6] Andersson H, Ekblom B, Krustrup P. Elite football on artificial turf versus natural grass: movement patterns, technical standards, and player impressions. J Sports Sci. 2008;26(2):113–22. doi: 10.1080/02640410701422076 17852688

[7] Modric T, Esco M, Perkovic S, Basic Z, Versic S, Morgans R, et al. Artificial turf increases the physical demand of soccer by heightening match running performance compared with natural grass. J Strength Cond Res. 2023;37(11):2222–8. doi: 10.1519/JSC.0000000000004539 37883399

[8] Hales ME, Johnson JD. The influence of sport-field properties on muscle-recruitment patterns and metabolic response. Int J Sports Physiol Perform. 2019;14(1):83–90. doi: 10.1123/ijspp.2018-0004 29893589

[9] Hales M, Johnson JD. The influence of time-dependent surface properties on sprint running performance between male and female athletes. IJKSS. 2020;8(4):42. doi: 10.7575/aiac.ijkss.v.8n.4p.42

[10] Kutnjak M, Pavlinovic V, Modric T. The effect of pitch surface on match running performance in women’s soccer. Appl Sci. 2024;15(1):40. doi: 10.3390/app15010040

[11] Lopes H, Carrilho D, Vaz de Carvalho M, Brito H, Carvalho A, Araújo D. The role of eco-physical variables for analyzing and modeling goal-directed behavior in sport. Acta Psychol (Amst). 2025;261:105826. doi: 10.1016/j.actpsy.2025.105826 41138593

[12] FBref.com (Sports Reference LLC). Major League Soccer Stats [Internet]. Available from: https://fbref.com/en/comps/22/Major-League-Soccer-Stats

[13] San Jose Earthquakes. New SISGrass hybrid field coming to Earthquakes Stadium [Internet]. Available from: https://www.sjearthquakes.com/news/news-new-sisgrass-hybrid-field-coming-earthquakes-stadium

[14] Diaz-Cidoncha Garcia J, Alvarez EF, Dellal A. Analysis of offensive technical actions on different playing surfaces in 5v5, 7v7, and 9v9 sided games in soccer. Int J Sports Phys Educ. 2017;3(4):47–57. doi: 10.20431/2454-6380.0304008

[15] Chacón-Fernández E, Brunsó-Costal G, Duarte A, Sánchez-Oro J, Alonso-Pérez-Chao E. Home advantage in football: exploring its effect on individual performance. Appl Sci. 2025;15(4):2242. doi: 10.3390/app15042242

[16] Krumer A, Shapir O, Zou Y. The size of the crowd and home advantage in football. Asian J Sports Exerc Psychol. 2024;4(3):82–7. doi: 10.1016/j.ajsep.2024.10.009

[17] Rogerson M, Knight D, Scherer R, Jones B, McManus C, Waterworth S, et al. Meta-analysis of the effects of VAR on goals scored and home advantage in football. Proc Inst Mech Eng P: J Sports Eng Technol. 2024;240(1):283–91. doi: 10.1177/17543371241242914

[18] Hvattum LM. Playing on artificial turf may be an advantage for Norwegian soccer teams. J Quant Anal Sports. 2015;11(3):182–92. doi: 10.1515/jqas-2014-0046

[19] Travassos B, Davids K, Araújo D, Esteves TP. Performance analysis in team sports: advances from an Ecological Dynamics approach. Int J Perform Anal Sport. 2013;13(1):83–95. doi: 10.1080/24748668.2013.11868633

[20] Kubayi A, Toriola A. The influence of situational variables on ball possession in the South African Premier Soccer League. J Hum Kinet. 2019;66:175–81. doi: 10.2478/hukin-2018-0056 30988851 PMC6458569

