# Supplementary material for: Playing surface, match outcomes, and formation usage patterns in Major League Soccer
Source: PLoS One. 2026 Sep 18;21(9):e0358364. doi: 10.1371/journal.pone.0358364 (PMC13588534; doi:10.1371/journal.pone.0358364)
Supplement: Supplementary file 1 [file pone.0358364.s002.docx]

| **n** | **What it represents** | **Where it is used** |
| --- | --- | --- |
| **6,764** | Total team-match observations in the cleaned dataset. Each match is represented by two team-match records (one per team); reflects removal of one duplicate entry and one record for a match cancelled in 2020. | Base denominator for the dataset as a whole; Abstract, Study Design. |
| **3,383** | Total distinct matches, derived by retaining one team-match record per match (home-team perspective). This deduplication is also the basis for the primary test of match outcome by surface type (Hypothesis 1), since each match’s two team-match records report the identical outcome from opposite perspectives. | Reported wherever the manuscript describes a count of matches rather than team-match observations; Hypothesis 1. |
| **6,761** | Team-match observations with an identifiable playing surface (subset of n = 6,764); 3 team-match observations lack a surface classification and are excluded. | Denominator for Table 2 and Table 3. |
| **3,383** | Distinct matches with an identifiable playing surface. Identical to the total distinct-match count above because every distinct match in the dataset has an identifiable surface. | Abstract and Study Design sample-size definitions. |
| **5,030 / 1,477 / 254** | Team-match observations by surface type—grass, turf, hybrid (subsets of n = 6,761; sum to 6,761). | Table 2’s n column; denominator for Table 3’s formation percentages. |
| **4,991 / 1,457 / 254** | Team-match observations by surface type with usable (non-shootout) goals data (subsets of the row above). | Goals For/Against statistics in Table 2; the goals-scored ANOVA. |
| **59** | Team-match observations excluded from goals-based analyses because their recorded score used penalty-shootout notation (e.g., “0 (1)”) rather than a plain numeric value. | Applies only to Goals For/Against (Table 2) and the associated ANOVA; possession and outcome statistics are unaffected. |
| **F(2, 6699)** | Degrees of freedom for the goals-scored ANOVA and the goal-margin ANOVA, based on 6,702 valid goal observations (4,991 + 1,457 + 254) across three surface groups. | Results, Performance Metrics. |
| **95** | Team-match observations from the 2020 'MLS is Back' tournament, played entirely at a neutral site (47 home-designated records, 48 away-designated records). The asymmetry reflects forfeited group-stage matches involving two clubs, FC Dallas and Nashville SC, that withdrew from the tournament due to COVID-19 outbreaks; these matches are recorded only from their opponents' perspective, which need not preserve an even home/away split. Of these 95 observations, 3 lack an identifiable surface — the same 3 observations noted above as missing surface classification — leaving 92 tournament observations with identifiable surface. | Retained in surface-based analyses; excluded from all analyses involving match location. |
| **6,668** | Final analytic sample for Table 1, derived by excluding the 92 tournament observations and 1 further observation with incomplete performance data from the 6,761 team-match observations with identifiable surface. | Table 1; the surface × location outcome-distribution test. |
| **6,669** | Team-match observations used for the overall test of home-pitch advantage independent of surface. Excludes only the 95 neutral-tournament observations; does not require an identifiable surface. | Overall home-advantage χ² test (Results, Surface-Related Analyses). |
| **6,562** | Consecutive within-season, within-team match transitions (pairs of a team’s successive matches with valid surface and formation data) used to test whether formation changes coincide with surface changes. Unlike the sample sizes above, this is a count of match-to-match pairs, not individual team-match observations: of the 6,760 team-match observations with both surface and formation available, each of the 198 distinct team-season groupings contributes one fewer transition than its match total, since a team’s first match of a season has no preceding match within that season to compare against (6,760 − 198 = 6,562). | Formation-change-on-surface-change analysis (Results, Formation Usage). |
