# Supplementary material for: Playing surface, match outcomes, and formation usage patterns in Major League Soccer
Source: PLoS One. 2026 Sep 18;21(9):e0358364. doi: 10.1371/journal.pone.0358364 (PMC13588534; doi:10.1371/journal.pone.0358364)
Supplement: Supplementary file 2 [file pone.0358364.s003.docx]

| **Formation Family** | **Included Recorded Formations** |
| --- | --- |
| *Back Four-Three-Forward Family* | 4–3–3 |
| *Back Four-Double-Pivot Systems* | 4–2–3–1; 4–1–4–1; 4–5–1 |
| *Back Four-Two-Forward Systems* | 4–4–2; 4–4–1–1; 4–2–2–2; 4–2–2–1–1 |
| *Back Four-Narrow-Diamond Systems* | 4–1–2–1–2; 4–3–1–2; 4–1–3–2 |
| *Back Three-Wingback Systems* | 3–4–3; 3–4–2–1; 3–4–1–2; 3–1–4–2 |
| *Back Three-Midfield-Heavy Systems* | 3–5–2; 3–5–1–1; 3–3–3–1; 3–2–1–2–2;   3–3–2–2; 3–2–3–2 |
| *Back Five – Defensive Variants* | 5–3–2; 5–4–1 |
| *Hybrid / Asymmetric Back Lines* | 3–2–2–1–2; 3–2–2–2–1 |
