# Supplementary material for: Playing surface, match outcomes, and formation usage patterns in Major League Soccer
Source: PLoS One. 2026 Sep 18;21(9):e0358364. doi: 10.1371/journal.pone.0358364 (PMC13588534; doi:10.1371/journal.pone.0358364)
Supplement: Supplementary file 3 [file pone.0358364.s004.docx]

| **Team** | **Home Pitch Surface** |
| --- | --- |
| Atlanta United | Turf |
| Austin | Grass |
| CF Montreal | Grass |
| Charlotte | Turf |
| Chicago Fire | Grass |
| Colorado | Grass |
| Columbus | Grass |
| DC United | Grass |
| FC Cincinnati | Turf |
| FC Dallas | Grass |
| Houston | Grass |
| Inter Miami | Grass |
| LA Galaxy | Grass |
| LAFC | Grass |
| Minnesota | Grass |
| New England Revolution | Turf |
| NY Red Bulls | Grass |
| NYCFC | Grass |
| Nashville SC | Grass |
| Orlando City | Grass |
| Philadelphia Union | Grass |
| Portland | Turf |
| Real Salt Lake | Grass |
| San Jose Earthquakes | Hybrid |
| Seattle | Turf |
| Sporting Kansas City | Grass |
| St. Louis | Grass |
| Toronto FC | Grass |
